# Volumetric Abnormalities in Violent Schizophrenia Patients on the General Psychiatric Ward

**DOI:** 10.3389/fpsyt.2020.00788

**Published:** 2020-08-28

**Authors:** FengJu Liu, Yang Shao, Xin Li, Li Liu, Rong Zhao, Bin Xie, Yi Qiao

**Affiliations:** ^1^Shanghai Mental Health Center, Shanghai Jiao Tong University School of Medicine, Shanghai, China; ^2^Shanghai Pudong New Area Mental Health Center, School of Medicine, Tongji University, Shanghai, China

**Keywords:** structural brain alterations, violence, schizophrenia, MRI voxel-based morphometry, neuroimaging

## Abstract

**Background:**

In recent years, neuroimaging has been used increasingly to explore the biological underpinnings of violence carried out by schizophrenia patients (SPs). Studies have focused mostly on patients with a history of carrying out severe physical assaults, or comorbid with substance abuse/personality disorder (SA/PD). As a result, participants were unrepresentative and the interpretation of brain-structure changes was confusing. Here, we concentrated on SPs on a general psychiatric ward with a history of relatively lower violence, and individuals comorbid with SA or PD were excluded. We expected to identify the characteristics of brain morphometry in this population, and to explore whether the morphometric changes were universal.

**Methods:**

Forty-eight violent schizophrenia patients (VSPs), twenty-seven non-VSPs (nVSPs) and 28 nonviolent healthy controls (HCs) were investigated. Voxel-based morphometry was used to evaluate the gray matter volume (GMV) of all study participants. Whole-brain analyses were used to reveal group effects and differences between any two groups. Correlation analyses were undertaken between significant brain regions and behavioral measurements in the VSP group.

**Results:**

Patients showed a significantly smaller GMV in widespread frontal, temporal, and limbic regions compared with HCs. No region was found in which the two patient groups had significantly larger volumes compared with that in HCs. A significant decrease in the GMV of the right fusiform gyrus was found in the VSP group compared with that in the nVSP group (p = 0.004), where the GMV of this region had a negative correlation with the Physical Aggression [subscale of the Modified Overt Aggression Scale (MOAS)] or Hostility score. The VSP group showed a trend of GMV decrease in the left middle temporal cortex compared with that in the nVSP group (p = 0.077). Negative correlation was also found between the GMV of left inferior temporal gyrus/left Superior frontal gyrus, medial and the Hostility score.

**Conclusions:**

Our results provide initial evidence demonstrating the generalizability of GMV abnormalities in SPs engaged in varying levels of violence, even when SA or PD have not been implicated. GMV reduction was correlated with only the Physical Aggression subscale score of the MOAS, suggesting that this change in brain morphology may be dependent upon different types of violent actions.

## Introduction

There is robust evidence that people with schizophrenia are more likely to carry out violence than members of the general population (1, 2). Such violence can result in physical harm, stigmatization, and enormous economic cost to society (3, 4). Therefore, it is important to develop a better understanding of the aggression and violence involved in schizophrenia. In addition, neurological soft signs have been shown to be present in violent schizophrenia patients (VSPs) compared with non-VSPs (nVSPs) and normal populations (5, 6), which suggests that neuropathologic predispositions contribute to aggression in schizophrenia.

Certain brain regions have important roles in the violent behavior associated with schizophrenia (7). These are regions involved in affective regulation, such as the orbital frontal cortex (OFC) and anterior cingulate cortex (ACC) (6, 8, 9) as well as egions involved in psychiatric symptoms, such as the hippocampus and prefrontal cortex (PFC) (10–12).

Several structural magnetic resonance imaging (MRI) studies have been conducted in VSPs. Hoptman and colleagues reported a correlation between the Urgency Score and reduced cortical thickness in the OFC and ACC (13). Similarly, the Impulsiveness Score was found to be correlated significantly and negatively with the gray matter volume (GMV) of the OFC and hippocampus in SPs with a history of severe violence (14). Few studies have focused on the relationship between brain structure and violence. Only a few studies have reported significant GMV differences among VSPs when compared with nVSPs. Yang and colleagues revealed that people convicted of murder and suffering from schizophrenia showed a reduced GMV in their hippocampus compared with that of patients without a history of violence (10). Kuroli and coworkers found reduced temporal, fusiform, and insular volumes in SPs who had carried out severe violence compared with nVSPS (15). In contrast, in a study undertaken in SPs who had carried out physical assaults, widespread morphometric abnormalities were found in VSPs compared with healthy controls (HCs), but shared volumetric abnormalities were not found between VSPs and nVSPs (16). Similarly, a reduced volume of the ACC was revealed in SPs who had carried out severe violence only when they were compared with HCs (17). In another study undertaken on SPs who had carried out severe violence, the volume differences in putamen and amygdala between VSPs and nVSPs disappeared when the influence of the Positive and Negative Syndrome Scale (PANSS) score was minimized (18).

Results between studies have been inconsistent, with some particular areas of concern: different definitions of violence; small sample size; comorbidity with substance dependence, personality disorder (PD) and other factors. Substance abuse and PD, as confounding factors, can affect violent behavior (19, 20), and might play an important part in morphologic changes (15, 18, 21), which can complicate interpretation of volumetric changes. Structural-MRI studies have been carried out mostly on SPs who have carried out severe physical violence (14, 15), near-fatal violent behavior against another person (18) or murder (10). These are extreme cases of SPs, so these participants are unrepresentative.

With the limitations mentioned above in mind, we concentrated on SPs on a general psychiatric ward with a history of relatively low-level violence, and individuals comorbid with substance abuse or PD were excluded. We used voxel-based morphometry to detect neural differences in VSPs, nVSPs and non-violent HCs to explore the neurobiological basis underlying these categories of the population.

## Methods

### Ethical Approval of the Study Protocol

All participants and/or their authorized legal representatives provided written informed consent. The study protocol was approved by the Ethics Committee of Shanghai Mental Health Center (Shanghai, China).

### Participants

The study cohort comprised 75 SPs and 28 HCs. SPs were recruited on the general psychiatry ward of Shanghai Mental Health Center (Shanghai, China). All patients met ICD-10 diagnostic criteria, including 48 SPs who had a history of violence and ultimately 27 age-matched nVSPs. One participant dropped out of the nVSP group for not being able to tolerate MRI. All patients were medicated. In the VSP group, 30 patients were on atypical agents, one was on typical agents, and 17 were on both types of agents. In the nVSP group, 17 patients were on atypical agents, one was on typical agents, and nine were on both types of agents. None of the patients had a history of substance dependence. Urine tests were undertaken when patients were admitted to the ward. HCs were recruited from the local community. Diagnostic interviews were conducted by two independent psychiatrists to screen for a history of mental disorders. HCs were screened for PD using the Structured Clinical Interview for DSM-IV Axis II Disorders (SCID-II). Self-reporting indicated that no patients had a history of substance abuse. All participants were required to be aged 18–45 years and right-handed to minimize the influence of hemispheric lateralization between three groups. Participants with severe physical diseases, head injuries, or diagnosed with other psychiatric disorders were excluded.

### Clinical Measurements

Psychiatric symptoms in patients were rated using the PANSS. We used the Modified Overt Aggression Scale (MOAS) to evaluate acts of violence, as noted previously (22, 23). The MOAS is composed of four subscales: Verbal Aggression; Aggression Against Objects; Physical Aggression Against Oneself; Physical Aggression Against Other People. This scale has good reliability and validity, and is also applicable to Chinese populations (24, 25).

All individuals in the VSP group were required to have a MOAS score >4 in the past 6 months. Otherwise the participants were classified into the nVSP group. In the PANSS, the Excitement item (P4), Hostility item (P7) and Poor Impulse Control item (G14) were also used to describe aggressive behavior (26).

### Acquisition and Preprocessing of Images

All participants were images on the same Magnetom Verio 3.0-T MRI scanner (Siemens, Munich, Germany) at Shanghai Mental Health Center. The T1-weighted sequence was scanned with the parameters of repetition time = 2530 ms, echo time = 2.34 ms, inversion time = 1100 ms, flip angle = 7°, field of view = 256 mm, with 192 1-mm slices (no gap).

Data were processed using Statistical Parametric Mapping (SPM8) (www.fil.ion.ucl.ac.uk/spm) and voxel-based morphometry (VBM8) toolbox with default parameters (http://dbm.neuro.uni-jena.de/vbm/) in Matlab 2012a. Structural T1-weighted MR images were bias-corrected and segmented into gray matter (GM), white matter, and cerebrospinal fluid. Images of GM and white matter were normalized spatially to the standard Montreal Neurological Institute template and modulated to account for the volume changes caused by normalization. The modulated GM images were smoothed further with a Gaussian kernel of 8-mm full-width-at-half maximum.

### Statistical Analyses

#### Demographic and Behavioral Data

Analyses were undertaken with SPSS 18.0 (IBM, Armonk, NY, USA). Continuous variables were analyzed using one-way analysis of variance (ANOVA) with *post hoc* comparisons of the mean (least square difference method) or independent *t*-test, as appropriate. The chi-square test was used to examine categorical variables.

#### Data on Whole-Brain Structure

Differences in the GMV were analyzed by a one-way ANOVA in the three groups controlling for the whole-brain GMV and education level. A whole-brain F-test was undertaken to determine differences among the three groups. Then, significant clusters were saved and extracted as region of interest (ROI) “masks.” The mean value of a cluster for each participant was calculated for *post hoc* analysis to reveal GMV differences between any two groups.

For the whole-brain F-test, the familywise error (FWE) was corrected using a voxel-level correction implemented in SPM8 and the significance level was set at corrected p < 0.05 with a minimum of 10 voxels. For *post hoc* analyses, the significance level was set at p < 0.05.

#### Correlation Analyses

In the VSP group, correlation analyses were carried out between significant brain regions according to whole-brain analyses and behavioral measurements. Spearman’s correlation (using SPSS v18.0) was used for these steps.

## Results

### Comparisons of Demographic Statistics

As reported in **Table 1**, no significant group differences existed for age (F = 1.565, p = 0.214) or sex (c^2^ = 0.463, p = 0.793), with the sample being comprised mainly of men. Groups differed in terms of years of education (F = 10.648, p < 0.000), with HCs having spent more years at school than the nVSP group and VSP group. When comparing VSPs and nVSPs, no significant group differences existed for illness duration (t = 0.762, p = 0.449). The medication dose (as measured using chlorpromazine equivalency values) did not differ (t = 0.589, p = 0.746) (27). All patients were taking antipsychotic medications during the study. As expected, VSPs showed significantly higher scores on the MOAS than nVSPs and HCs (F = 191.242, p < 0.000). No significant differences in the negative score or total score of the PANSS were found in VSP and nVSP group. However, VSP group had higher scores on the PANSS positive scale, this mainly because VSP group had higher rating on items measuring violent traits.

**Table 1:** Demographic and clinical characteristics of the study groups.

	Mean (SD)			F/χ^2^/T	P
	VSP(n = 48)	nVSP(n = 27)	HC(n = 28)		
Age, years	28.98 (7.85)	27.70 (6.41)	26.07 (5.57)	1.565	0.214
Education, years	12.90 (2.81)	13.11 (2.82)	15.57 (1.64)	10.648	**0.000**
MOAS score	14.10 (5.12)	0.37 (0.79)	0.36 (0.83)	191.242	**0.000**
Age at illness onset, years	21.42 (5.81)	21.52 (5.02)		−0.076	0.939
Illness duration, years	7.17 (4.71)	6.23 (5.80)		0.762	0.449
Medication dose (chlorpromazine equivalent)	539.42 (275.56)	500.30 (277.06)		0.589	0.746
PANSS total score	84.65 (13.59)	83.78 (9.51)		0.294	0.770
PANSS positive score	25.00 (4.98)	21.30 (4.29)		3.245	**0.002**
PANSS negative score	17.13 (6.14)	19.89 (5.26)		−1.976	0.053
Sex (M/F)	32/16	20/7	19/9	0.463	0.793

VSP, violent schizophrenia patient; nVSP, nonviolent schizophrenia patient; HC, healthy control; PANSS, Positive and Negative Symptom Scale; MOAS, Modified Overt Aggression Scale. The results for P < .05 were marked in bold.

### Whole-Brain Analyses and Post Hoc Comparisons

**Figure 1:**
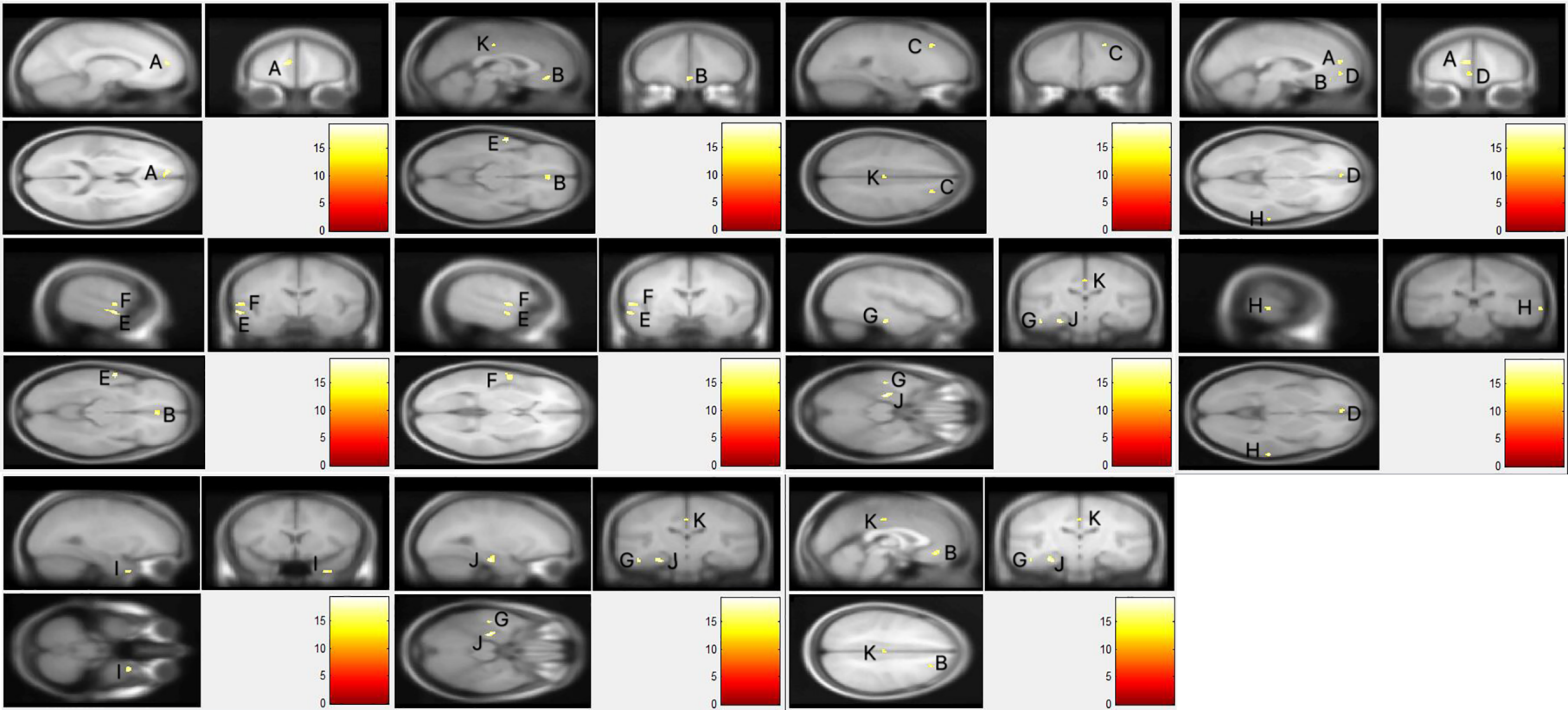
The whole brain gray matter volume differences of three groups. Using F-test, the familywise error (FWE) was corrected using a voxel-level correction and the significance level was set at corrected p < 0.05 with a minimum of 10 voxels. **(A)** Left Superior frontal gyrus, medial; **(B)** left/right middle frontal gyrus, orbital part; **(C)** right middle frontal gyrus; **(D)** left superior frontal gyrus, medial orbital; **(E)** left middle temporal gyrus; **(F)** left superior temporal gyrus; **(G)** left inferior temporal gyrus; **(H)** right middle temporal gyrus; **(I)** right fusiform gyrus; **(J)** left parahippocampal gyrus/left hippocampus; **(K)** left median cingulate gyrus.

As presented in **Figure 1**, voxel-wise whole-brain analyses showed significant GMV differences in widespread brain regions between three groups (FWE-corrected p < 0.05). These regions were: (i) frontal regions of bilateral orbital part of middle frontal gyri, left medial of the superior frontal cortex, and right middle frontal cortex; (ii) temporal regions of bilateral middle temporal gyri, left superior temporal cortex, left inferior temporal cortex, and right fusiform gyrus; (iii) limbic system of the left parahippocampus, and hippocampus, and left middle cingulate gyrus. Descriptive statistics for these brain regions of the three groups and *post hoc* results are presented in **Tables 2** and **3**. Details were shown in **Data Sheet 1** and **Data Sheet 2** in the supplementary data.

**Table 2:** *Post hoc* analyses (differences between any two groups).

Anatomic Location	MNI peak coordinate			Voxels		F		P		VSP vs. nVSP			VSP vs. HC				nVSP vs. HC		
	x	y	z					FWE- corrected		T		P	T		P		T		P
**Frontal lobe**																			
Left Superior frontal gyrus, medial	−11	54	13		134		19.432		0.002		−0.797	0.432	−6.137		**0.000**	−4.699		**0.000**	
Left /right middle frontal gyrus, orbital part	0	38	−12		86		17.305		0.009		0.813	0.409	−5.363		**0.000**	−5.453		**0.000**	
Right middle frontal gyrus	24	33	40		29		16.905		0.011		0.554	0.579	−5.358		**0.000**	−5.218		**0.000**	
Left superior frontal gyrus, medial orbital	−3	51	−5		28		15.883		0.022		0.438	0.657	−5.301		**0.000**	−5.065		**0.000**	
**Temporal lobe**																			
Left middle temporal gyrus	−60	−6	−11		90		19.250		0.002	−1.750		0.077	−6.894	**0.000**			−4.517	**0.000**	
Left superior temporal gyrus	−59	−4	3		80		18.500		0.004	−1.088		0.282	−6.285	**0.000**			−4.571	**0.000**	
Left inferior temporal gyrus	−47	−21	−24		39		17.499		0.008	−1.350		0.183	−5.946	**0.000**			−4.099	**0.000**	
Right middle temporal gyrus	68	−28	−3		26		17.305		0.009	−1.474		0.144	−6.110	**0.000**			−4.073	**0.000**	
Right fusiform gyrus	30	12	−45		47		17.250		0.009	−2.923		**0.004**	−6.000	**0.000**			−2.683	**0.000**	
**Limbic system**																			
Left parahippocampal gyrus /left hippocampus	−27	−21	−24		106		19.346		0.002		−0.887	0.346	−5.504	**0.000**			−4.062	**0.000**	
Left median cingulate gyrus	0	−19	40		13		15.628		0.027		0.731	0.467	−5.028	**0.000**			−5.085	**0.000**	

VSP, violent schizophrenia patient; nVSP, nonviolent schizophrenia patient; HC, healthy controls; peak voxel significant at P < 0.05; corrected for multiple comparisons after Family Wise Error (FWE). The results for P < .05 were marked in bold.

**Table 3:** Mean volumes of the ROI-defined gray-matter regions and their correlations with aggression.

Anatomic Location	MNI peak coordinate			VSP	nVSP	HC	Hostility		Physical Aggression Against Other People	
	x	y	z		Mean (SD)		rho	P	rho	P
**Frontal lobe**										
Left Superior frontal gyrus, medial	−11	54	13	0.52(0.06)	0.53(0.06)	0.61(0.06)	−0.285	0.050*	−0.097	0.512
Left/right middle frontal gyrus, orbital part	0	38	−12	0.72(0.07)	0.70(0.09)	0.82(0.07)	−0.087	0.557	−0.193	0.190
Right middle frontal gyrus	24	33	40	0.58(0.08)	0.57(0.07)	0.69(0.10)	−0.036	0.811	0.215	0.142
Left superior frontal gyrus, medial orbital	−3	51	−5	0.53(0.06)	0.52(0.07)	0.61(0.06)	−0.113	0.446	0.076	0.607
**Temporal lobe**										
Left middle temporal gyrus	−60	−6	−11	0.55(0.05)	0.57(0.07)	.65(0.06)	−0.144	0.329	−0.126	0.394
Left superior temporal gyrus	−59	−4	3	0.48(0.06)	0.50(0.06)	0.57(0.07)	−0.202	0.169	−0.086	0.562
Left inferior temporal gyrus	−47	−21	−24	0.49(0.07)	0.51(0.07)	0.59(0.08)	−0.347	0.016*	−0.232	0.112
Right middle temporal gyrus	68	−28	−3	0.50(0.06)	0.52(0.05)	0.58(0.05)	−0.186	0.205	−0.123	0.405
Right fusiform gyrus	30	12	−45	0.58(0.14)	0.67(0.13)	0.76(0.11)	−0.365	0.011*	−0.382	0.007*
**Limbic system**										
Left parahippocampal gyrus/left hippocampus	−27	−21	−24	0.77(0.05)	0.78(0.06)	0.83(0.05)	−0.006	0.968	0.042	0.778
Left median cingulate gyrus	0	−19	40	0.70(0.07)	0.70(0.07)	0.78(0.06)	−0.033	0.823	−0.010	0.944

Hostility, PANSS hostility item (P7); physical aggression against other people, fourth subscale of MOAS; ROI, regions of interest. *P < .05. The results for P < .05 were marked in bold.

Comparison of the nVSP vs. HCs (**Table 2**) revealed that nVSPs exhibited a volume decrease, relative to HCs, in all of the regions mentioned in the group comparisons. No region was found in which the nVSP group had a significantly larger volume compared with that of HCs.

When compared VSPs vs. HCs, the VSP group showed a smaller GMV compared with that of HCs in regions with significant group effects. Overall, the VSP group showed a more pronounced decrease in the GMV in most brain regions. We did not find a brain region in which the VSP group showed a larger GMV than that in HCs.

In addition, we compared VSPs vs. nVSPs. A significant decrease in the GMV of the right fusiform gyrus was observed in the VSP group compared with that in the nVSP group (p = 0.004). A decreased GMV in the left middle temporal gyrus in the VSP group was noted compared with that in the nVSP group (p = 0.077). We did not find a brain region in which the VSP group showed a larger GMV than that in nVSPs.

### Correlation Analyses

**Figure 2:**
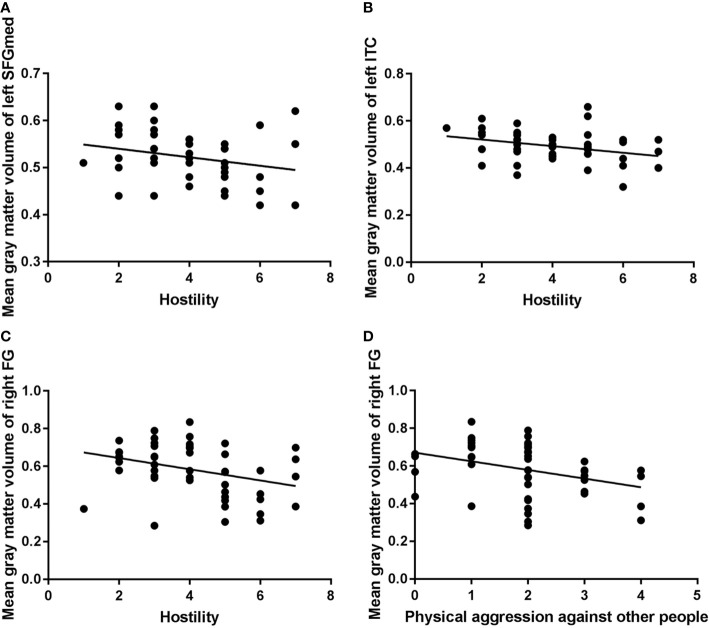
Correlation analysis of mean gray-matter volume of significant brain regions and aggressive behavior in the violent schizophrenia patient (VSP) group (n = 48). ITC, inferior temporal cortex; FG, fusiform gyrus; SFGmed, superior frontal gyrus, medial; P7, PANSS Hostility item. Negative correlations were seen between left SFGmed and P7 [**(A)**: rho = −0.285, P = 0.050)], left ITC and P7 [**(B)**: rho = −0.347, P = 0.016)], right FG and P7 [**(C)**: rho = −0.365, P = 0.011)], right FG and Physical Aggression Against Other People (subscale of the MOAS) [**(D)**: rho = −0.382, P = 0.007)] in the VSP group.

In the VSP group, Spearman correlation analyses showed a significant negative association between scores of Physical Aggression Against Other People (the fourth subscale of the MOAS) and volume of the right fusiform. Negative correlations were also seen between the Hostility item scores of the PANSS and the volumes of left inferior temporal, right fusiform, and the left medial of superior frontal GM (**Table 3** and **Figure 2**).

## Discussion

In our study, a decreased GMV in a widespread network of frontotemporal and limbic systems was detected in SPs compared with HCs. When considering violent or violent traits, the volume of the right fusiform was significantly smaller in the VSP group compared with that in the nVSP group and HCs. The volume decrease in this brain region appeared to be associated with physical aggression (subscale of the MOAS) and hostility. The VSP group also showed a trend in GMV decrease in the left middle temporal gyrus compared with that in the nVSP group. Negative correlation was also found between the GMV of several brain regions and behavioral measurements. These findings revealed different and shared abnormalities compared with studies on severely violent SPs (10, 14, 15, 18).

### Frontal Lobe

The VSP group and nVSP group showed a significantly decreased GMV in the bilateral medial of orbital frontal gyri, left medial of superior frontal gyrus, and right middle of frontal gyrus, compared with that in HCs. Similar abnormalities have been reported in SPs who have carried out severe physical violence. Compared with HCs, VSPs showed a reduced GMV and cortex thickness in the OFC and inferior frontal cortex (14, 28). The PFC plays an important part in the inhibitory control of aggression (29). GM abnormalities in the OFC and ventrolateral prefrontal cortex are considered to be associated with information-processing (30), decision-making (31), and emotion-regulation (9) mechanisms in violent behavior. We did not observe a significant difference in the GMV in frontal regions between VSPs and nVSPs, but noted a negative correlation between the volume of the superior frontal gyrus, medial and Hostility score in the VSP group. In accordance with our study, most studies have failed to find significant differences at the group level in the GMV of prefrontal regions when comparing VSPs with nVSPs (15, 18, 32). These observations might be because structural abnormalities in prefrontal regions seem to be more characteristic of schizophrenia or other violent traits than the violent act itself: violent behavior is perhaps more likely to result from a combination of several risk factors, including psychosis.

### Temporal Gyrus

We revealed brain-structure abnormalities in the temporal lobe (including the bilateral middle temporal gyri, left superior temporal cortex and inferior temporal cortex) in the two patient groups when compared with that in HCs. Consistent with our study, structural abnormalities in the temporal lobe have been reported in SPs (33) as well as in populations with psychopathy or antisocial behavioral problems (18, 34, 35). We did not find a volume difference in temporal regions between VSPs and nVSPs, but a negative correlation was found between the left inferior temporal cortex and the Hostility score. Contrary to our study, Kuroki and colleagues reported a reduced GMV in the right inferior gyrus in male SPs with a history of severe violence compared with that in nVSPs (15). Those differences were explained (at least in part) by varying levels of violence and other violent traits. In our study, violent behavior was mild, and correlations were found between the GMV and behavioral measurements instead of brain-structure differences. The temporal lobe is an important component of frontotemporal circuitry. It is involved in emotional processing and executive cognitive function, including the ability to use feedback to modulate behavior (36).

### Limbic Systems

A decreased GMV was observed in limbic systems, such as the left parahippocampal gyrus, hippocampal gyrus, and left middle cingulate gyrus, in the two patient groups compared with that in HCs. Similar to our findings, a reduced hippocampus volume was found in two studies of SPs with a history of severe physical assault (14, 18) compared with that in HCs. A reduced ACC volume and cortical thickness have been observed in SPs with a history of severe violence when compared with that in HCs (17, 28). A GMV difference in the hippocampal gyrus has been found between VSPs and nVSPs in several studies: those data are inconsistent with our study results. When compared with nVSPs, people convicted of murder and diagnosed with schizophrenia have exhibited a significant reduction in the GMV in the bilateral hippocampus (10). Such data indicate that volume changes in the hippocampus are dependent on the degree of violence. Abnormalities in the hippocampus may contribute toward neuropsychological disturbances and violence in schizophrenia. The hippocampus and ACC are critical components of the limbic system, and are involved in the regulation of violent behavior (30). The hippocampus is also involved in cognition, emotion, and regulation of the stress response (37). Structural abnormalities in the hippocampus may contribute to affective dysregulation and promote impulsive behavior which, in turn, might lead to higher chance of aggression (30).

### Fusiform Gyrus

VSPs showed a decreased GMV in the right fusiform compared with that in nVSPs. Similar findings were seen in two recent studies on SPs with a history of severe violence. Del Bene and colleagues revealed a reduced GMV in the right fusiform in SPs with a history of severely violent behaviors against others (15) compared with that in nVSPs. Storvestre and collaborators demonstrated that VSPs had a reduced thickness in the fusiform gyrus compared with that in nVSPs (38). Those findings demonstrated that the fusiform gyrus plays an important part in varying degrees of violence. The fusiform gyrus is part of the visual system, and has been proved to be involved in brain activity during anger-induced imagery tasks (39). Together with amygdala and other brain regions, the fusiform gyrus participates in processing affective visual stimuli in humans, contributing to the evaluation of which stimuli should be approached and which should be avoided (15, 40). This region is also part of the ventral stream, which is involved in facial recognition (41) and recognition of emotional stimuli (42).

Here we reported, for the first time, that the right fusiform gyrus has a close correlation with carrying out violent acts. One possibility why this has not been reported before is that previously scholars have chosen the OFC, PFC, amygdala, and hippocampus as ROI; the fusiform gyrus has often been excluded in such analyses. Future research should focus on the fusiform gyrus to ascertain the role of this brain region in violent behavior. Interestingly, in our study, the GMV of the right fusiform gyrus was correlated negatively only with physical aggression against other people, not with the total score or other subscale scores of the MOAS, which indicated the violence-specificity of this association. One reason for this finding could be that the neuropsychological traits of violence in SPs may be heterogeneous. Brain structure varies depending on the type of violent behavior carried out by SPs. For instance, physical aggression is associated with impulsivity, callousness, and lack of empathy, whereas suicide is caused by impairments of cognitive control of mood, pessimism, reactive aggressive traits, and excessive emotional pain (43–45).

### Strengths of Our Study

Unlike most studies on individuals with a history of severe violence, we focused on patients on a general psychiatry ward with a history of relatively lower severity of violence. In addition, patients in our study were free of substance abuse and PD, which simplified the interpretation of results. Therefore, the characteristics of participants in our investigation contributed to the study on violent behaviors in SPs. We were able to identify alterations in structure brain in VSPs on a general psychiatry ward so as to provide neuroimaging information for early recognition of violent behaviors in SPs.

### Limitations of Our Study

Our study had four main limitations. First, participants in the VSP group and nVSP group were taking antipsychotic medications. Though no significant differences in the chlorpromazine-equivalent dose were found in the two patient groups, we could not totally eliminate the influence of a long duration of medication use. Second, the education duration in participants with and without a history of violence was significantly less than that in HCs. Although we used education as a covariate, the influence of a confounding factor may not have been eliminated fully. Third, we did not correct for multiple comparisons in the correlation analysis, which would increase the probability of a type-I error. Finally, the absence of a group of non-psychotic violent patients prevented us from assessing whether the results were explained by an effect of violence or by an interaction between psychosis and violence.

## Conclusion

Findings provide initial evidence demonstrating the generalizability of GMV abnormalities in schizophrenia patients engaged in varying levels of violence. In our study, abnormality in the right fusiform was seen in violent schizophrenia group compared with non-violent patients. Gray matter volume decrease of this brain region was also associated with physical aggression (subscale of the MOAS) and hostility scores, which revealed an important role of right fusiform in violent behavior. Further study on the neurobiological mechanisms underlying this population may contribute to early detection and intervention on aggressive behavior in schizophrenia patients on general psychiatry ward.

## Data Availability Statement

All datasets presented in this study are included in the article/supplementary material.

## Ethics Statement

The studies involving human participants were reviewed and approved by Ethics Committee of Shanghai Mental Health Center. The patients/participants provided their written informed consent to participate in this study.

## Author Contributions

FL, YS, BX, and YQ designed the study and wrote the protocol. FL undertook the statistical analysis and wrote the first draft of the manuscript. XL, LL, and RZ collected clinical data. All authors contributed to the article and approved the submitted version.

## Funding

This investigation was supported by grants from the Three-Year Action Plan for the Construction of Public Health System in Shanghai (GWIV-5; principal investigator, BX), Key Projects in the National Science & Technology Pillar Program during the Twelfth Five-Year Plan Period (2012BAK16B04; principal investigator, BX), and the top priority of National Natural Science Foundation of China (81302624; principal investigator, YQ).

## Conflict of Interest

The authors declare that the research was conducted in the absence of any commercial or financial relationships that could be construed as a potential conflict of interest.
