# Supplementary material for: Volumetric Abnormalities in Violent Schizophrenia Patients on the General Psychiatric Ward
Source: Front Psychiatry. 2020 Aug 28;11:788. doi: 10.3389/fpsyt.2020.00788 (PMC7493665; doi:10.3389/fpsyt.2020.00788)
Supplement: Supplementary file 2 [file DataSheet_2.pdf]

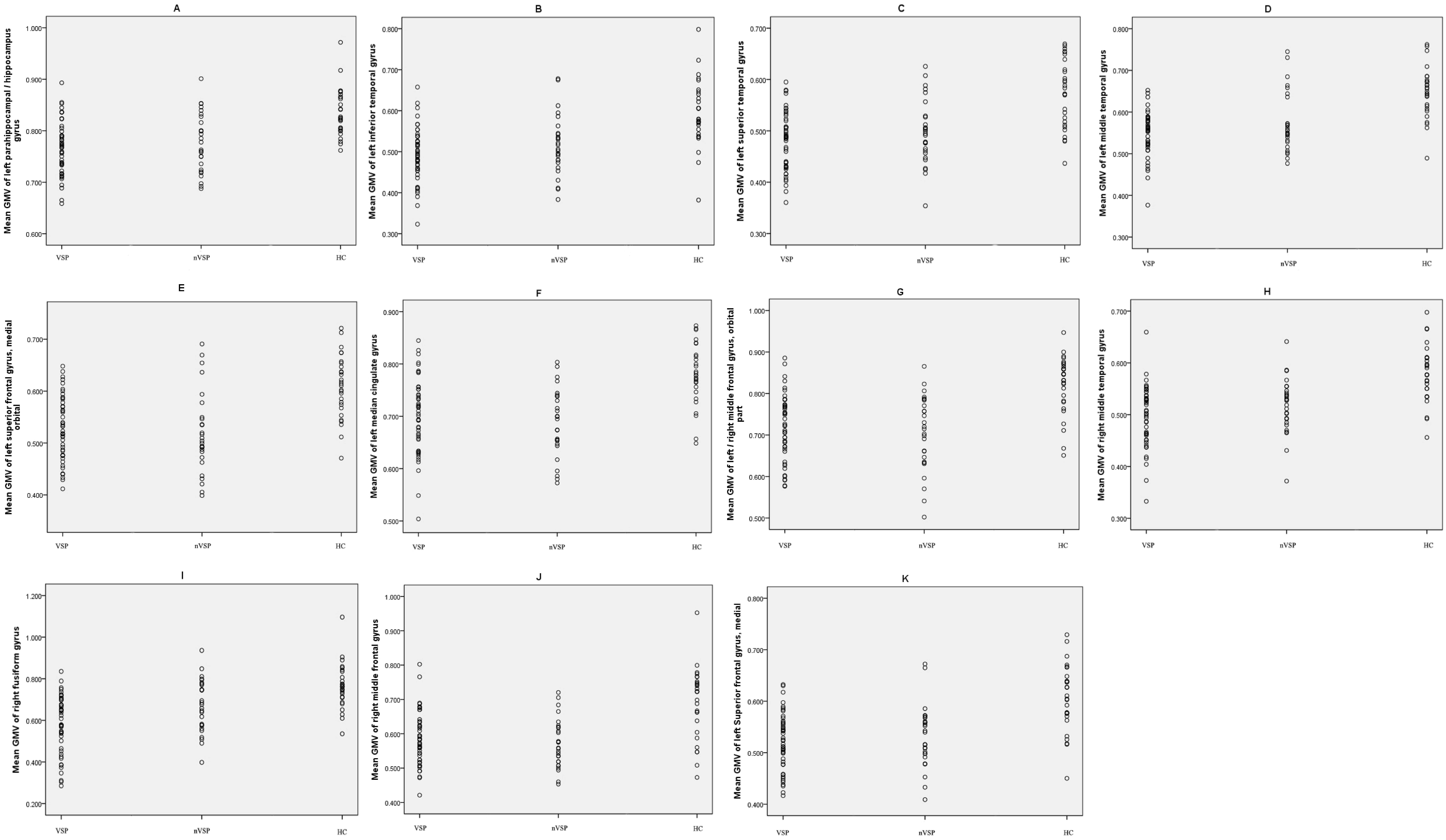

Fig. 3. Mean gray-matter volumes of significant brain regions in the violent schizophrenia patients (VSPs), non-violent schizophrenia patients (nVSPs) and healthy controls (HCs).
